# Changes in serum and urinary metabolomic profile after a dietary intervention in patients with irritable bowel syndrome

**DOI:** 10.1371/journal.pone.0257331

**Published:** 2021-10-11

**Authors:** Sanna Nybacka, Magnus Simrén, Stine Störsrud, Hans Törnblom, Anna Winkvist, Helen M. Lindqvist

**Affiliations:** 1 Department of Internal Medicine and Clinical Nutrition, Institute of Medicine, Sahlgrenska Academy, University of Gothenburg, Gothenburg, Sweden; 2 Department of Molecular and Clinical Medicine, Institute of Medicine, Sahlgrenska Academy, University of Gothenburg, Gothenburg, Sweden; 3 Center for Functional GI & Motility Disorders, University of North Carolina at Chapel Hill, Chapel Hill, North Carolina, United States of America; Oregon State University, UNITED STATES

## Abstract

**Background:**

Irritable bowel syndrome (IBS) is a multi-faceted gastrointestinal disorder where food intake often triggers symptoms. Metabolomics may provide mechanistical insights to why responses to dietary modifications are diverse.

**Objective:**

This study aimed to identify metabolite patterns related to dietary intake in patients with IBS, and to identify metabolites driving the separation between responders and non-responders to treatment.

**Methods:**

Participants were randomized to a low fermentable oligo-, di-, monosaccharide and polyol (FODMAP) diet (LFD) or traditional IBS diet (TID) for four weeks. Fasting serum and urine samples pre- and post-intervention were analyzed using ^1^H nuclear magnetic resonance (NMR) metabolomics. Response to treatment was defined as a reduction in IBS severity scoring system (IBS-SSS) ≥50.

**Results:**

Twenty-five individuals in the LFD (13 responders) and 28 in the TID (14 responders) were included in these post hoc analyses. In endpoint samples, significant decreases in polyols and glucose were seen in the LFD. Post-intervention samples revealed that LFD responders had significantly increased levels of 2-hydroxybuturate and decreased levels of glucose and pantothenic acid compared to non-responders. For the TID, only weak multivariate models were identified and a larger diversity in metabolite response compared to the LFD were noted.

**Conclusions:**

In this study, metabolite patterns between individuals who responded well to an LFD compared to non-responders could be distinguished. This provides new hypotheses for mechanistic actions related to response to dietary modifications, but the results need to be validated in larger cohorts.

**Clinical trial registration:**

This trial was registered at www.clinicaltrials.gov, registry number NCT02107625.

## Introduction

Irritable bowel syndrome (IBS) is one of the most common functional gastrointestinal (GI) disorders worldwide [1], characterized by recurrent abdominal pain together with altered stool frequency and/or stool form [2,3]. Symptoms of IBS are often closely related to food intake, and individual foods or meals are often reported to trigger or worsen GI symptoms [4–6]. The mechanisms of action are still largely unknown, but increasing evidence suggests that an altered gut microbiota composition can affect the digestion and metabolism of foods in an unfavorable way [7,8].

Whilst IBS is considered to be a chronic condition, the emphasis on the treatment is to reduce symptoms of IBS, rather than to cure the disease as we still lack treatment options that can influence the long-term evolution of the disorder. Dietary modifications are often encouraged as a first line treatment and traditional IBS diet (TID) has to a large extent focused on eating behavior, emphasizing portion size control and regular consumption, and limiting intake of foods believed to cause bloating (cabbage, onions, fizzy drinks etc) [9,10]. Recently, the role of fermentable carbohydrates (Fermentable Oligosaccharides, Disaccharides, Monosaccharides And Polyols, FODMAPs) for triggering GI symptoms has gained interest [11]. The low FODMAP diet (LFD) as a concept focuses on limiting intake of carbohydrates that are poorly absorbed in the small intestine. These include fructose and undigested lactose, which exert an osmotic action with an increased water retention, leading to a distention of the small bowel [12]. Also, when reaching the colon, these non-absorbed carbohydrates that also include oligosaccharides and polyols that pass undigested to the colon, are rapidly metabolized by the gut microbiota, causing a fermentation process where luminal gas is formed. The increased colon distention is likely to cause pain in IBS patients with visceral hypersensitivity [12].

Recently, our group performed a randomized diet intervention study with the aim of comparing a TID to an LFD in patients with IBS [13]. The study showed that approximately 50% of all patients achieved significant symptom reductions (defined as a reduction in the IBS-Symptom Severity Score; IBS-SSS of at least 50), regardless of intervention diet. However, little is still known about the mechanisms explaining why some individuals respond positively to dietary modifications, whereas symptoms will remain unaltered for others.

Modern metabolomics offers a possibility to measure small molecules, or metabolites, which are produced both endogenously and from the digestion and metabolism of foods. Metabolomics can capture the effect of both genetics and environment (gender, food intake, physical activity, medication etc) on metabolites in bio-fluids, such as serum or urine. A previous study has demonstrated differences in urine metabolites between a group of IBS patients receiving a low FODMAP diet compared to a high FODMAP diet, where an eightfold reduction in histamine levels were noted in the group receiving a low FODMAP diet, providing interesting hypotheses for pathophysiological mechanisms in IBS [14]. In this post hoc assessment of our previously published study, we aimed to study the serum and urinary metabolomic fingerprints of patients with IBS who participated in our dietary intervention trial [13], and to study if it was possible to characterize the change in metabolite concentrations according to the outcome of the dietary modifications. We hypothesized that the IBS patients’ baseline dietary intake would be reflected in their baseline metabolite profile, and that it would be possible to relate changes in symptom severity during the trial (i.e., being a responder or not) to changes in metabolite concentrations.

## Subjects and methods

### Study design and participants

These post hoc analyses were based on a randomized dietary intervention trial with the aim of comparing two dietary regimes in the management of IBS symptoms. Detailed information about the study and participants have previously been described in Böhn et al [13]. In short, women and men >18 years of age diagnosed according to the ROME III criteria for IBS [15] were consecutively recruited through outpatient clinics in Gothenburg and Stockholm, Sweden, between 26 September 2013 and 4 April 2014. Only patients reporting moderate to severe IBS symptoms, i.e. IBS-SSS≥175 [16] were eligible for inclusion. Intake of probiotics and/or a lactose-reduced diet was allowed as long as the intake was kept consistent throughout the whole study period. Patients were excluded if they had other GI diseases explaining their symptoms, other serious chronic diseases, severe psychiatric diseases, being pregnant, or if they were unable to reliably respond to questionnaires in Swedish. Patients were also not allowed to be on any nutrient restrictive diet prior to the study or to have any food allergies. Sample size of participants was determined by the main outcome in the study, i.e. to be able to detect a difference of ≥50 points in IBS-SSS by the two diets with two-sided α = 0.05 and 80% power. In accordance, at least 31 participants were needed in each group. This trial was registered at www.clinicaltrials.gov (NCT02107625).

### Study protocol

Before any data were assembled, all study participants provided their written informed consent. Procedures followed were approved by the regional Ethical Review Board in Gothenburg and in accordance with the Helsinki Declaration of 1975 as revised in 1983. Detailed information about the intervention and the procedure has previously been described in detail [13]. At the first visit, anthropometric and demographic data were collected, and a 10-day screening period was initiated. During the screening period a daily stool diary was completed for IBS subtyping [15]. Also, a four-day food record (Wednesday-Saturday) was filled in by the participants where all foods and drinks consumed were noted, and stool and urine samples were collected. At the second visit, baseline fasting blood samples were drawn. Patients reporting moderate to severe IBS symptoms, i.e. IBS-SSS≥175 were randomized with a 1:1 allocation to a four-week intervention with either TID, or LFD. During the intervention period subjects completed a daily stool form diary, and during the last week once more recorded their diet for four days and collected a stool and a urine sample. After finishing the intervention, patients returned for a last visit where the IBS-SSS was filled in and endpoint fasting blood samples were drawn.

### Sampling and sample pre-processing of serum and urine

Baseline serum samples were drawn at the randomization visit (visit 2) and at the end of the intervention (visit 3). Samples were drawn after an overnight fast, where no food was allowed after 10 P.M. and only a small amount of water was allowed to drink if needed. Four ml of serum was drawn (Z Serum Sep Clot Activator), rested for 45 minutes, and then centrifuged in 4°C at 3800 rpm for 10 minutes. Thereafter 500 μl was aliquoted into cryo vials and frozen in -80°C within 2 hours. Serum samples were thawed for 60 min at 4°C, 100 μL serum was mixed with 100 μL phosphate buffer (75 mM Na2HPO4, 20% D2O, 0.2 mM imidazole, 4% NaN3, 0.08% TSP-d4, pH 7.4) in a deepwell plate. Finally, 180μL sample mix was transferred to 3.0 mm NMR tubes (Bruker BioSpin, 96 sample racks for SampleJet) using a SamplePro L liquid handling robot (Bruker BioSpin, Rheinstetten, Germany). Samples were kept at 6°C in the SampleJet sample changer until analysis.

Ten ml of fasting urine samples were collected in the morning of visit 2 and visit 3. These were also centrifuged in 4°C at 3800 rpm for 10 minutes, before being aliquoted into 2 ml vials and frozen in -80°C. Prior to proton nuclear magnetic resonance (^1^H NMR) analysis, urine samples were thawed at 4°C overnight and centrifuged at 10000xg for 5 min. Then, 25 μl buffer (1.5 M KH2PO4 in D2O, 0.1% TSP-d4 and 0.5% NaN3, pD 6.95) was added to each well of a deepwell plate. Thereafter 225 μL of urine supernatant was transferred to the deepwell plate with a SamplePro L robot. The plate was then shaken at 400 rpm, 12°C for 5 minutes in an Eppendorf Thermomixer Comfort before transfer of 180 μl to 3.0 mm NMR tubes (Bruker BioSpin, 96 sample racks for SampleJet) using the SamplePro L liquid handler.

### NMR spectroscopy

^1^H-NMR spectra were measured at 800 MHz using Bruker Avance III HD spectrometer equipped with a 3 mm TCI cryoprobe and a cooled (6°C) SampleJet for sample handling. All ^1^H-NMR experiments were performed at 298 K. NMR data (1D perfect echo with excitation sculpting for water suppression) was recorded using the Bruker pulse sequence ’zgespe’ with (serum) or without (urine) a CPMG pulse train to suppress macromolecular resonances. The spectral width was 20 ppm, the relaxation delay 3 s, the acquisition time 2.04 s and a total of 128 scans were collected into 64k data points resulting in a measurement time for each sample of approximately 12 minutes. All data sets were zero filled to 128 k and an exponential line-broadening of 0.3 Hz was applied before Fourier transformation. All data processing was performed with TopSpin 3.2pl7 (Bruker BioSpin, Rheinstetten, Germany) and TSP-d4 was used for referencing.

Optimal buckets were performed using the MatLab function opt bucket [17] with size bucket = 0.04ppm and slackness = 0.5. The bucketed spectra were probabilistic quotient normalized [18] using an inhouse MatLab algorithm. Chenomx NMR suite 8.4 (Chenomx Inc., Edmonton, Canada) was used for annotation with the aid of the Human Metabolome Database (HMDB) [19] and an in-house implementation of the STOCSY routine [20].

### Data processing

^1^H-NMR spectra were aligned using icoshift [21] and manual integration of peaks was performed to a linear baseline on all spectra in parallel using an in-house MatLab (MathWorks, Natick, USA) routine. For serum, a total of 302 peaks were integrated within the chemical shift range of 0.829–8.449 ppm. Prior to integration of urine samples, the water region was removed. In total 499 peaks were integrated within the chemical shift range of -5.118–14.844 ppm. No sections of the spectra were excluded. Data for serum samples were UV-scaled and data for urine samples were pareto-scaled.

### Statistical analyses

For comparisons on baseline characteristics and diet intake, normally distributed data were analyzed by Student’s t-test (paired t-test within individuals and unpaired t-test between groups). Metabolite levels are generally not normally distributed and thus Wilcoxon signed rank exact test was used to evaluate variables pre vs. post-intervention, and Mann-Whitney U exact test for unpaired comparisons between responders vs. non-responders. Further, multivariable logistic regression was used to evaluate if metabolites that discriminated most between responders and non-responders in the OPLS-DA models remained significant also after adjustment for age and BMI. These analyses were performed using IBM SPSS Statistics for Windows, Version 22.0.0 (Armonk, NY: IBM Corp.).

### Multivariate methods

Principal component analysis (PCA) was used to explore clustering patterns of observations, trends in the data and outliers. In urine, three individuals were identified as outliers. These were explained by a total of eight buckets with unusual high loadings. Since two samples were analyzed for each individual, the values for these eight buckets were imputed with values from the other sampling. If one individual had the same extreme high loadings at both occasions, bucket values were imputed with group mean values for that bucket. An Orthogonal Projections to Latent Structures (OPLS) model was used to visualize the impact of clinical characteristics and dietary intake on metabolite profiles and models. Separation of classes and variables related to diet intervention or to response on dietary intervention were evaluated using Orthogonal Projections to Latent Structures Discriminant Analysis (OPLS-DA). The validity of the models was assessed by using cross validation of every 7th sample (default setting in SIMCA), permutation tests (n = 999) and Coefficient of Variation-Analysis of Variance testing (CV-ANOVA). Multivariate analysis yields an R^2^ value, which describes the fraction of the variation in the components that are captured by the model (the goodness of fit). A value of R^2^ = 1 indicates a perfect fit whereas a value >0.5 can be considered as a good fit. The Q^2^ value describes the predictive capacity of the model after cross-validation, and for biological samples a Q^2^ >0.4 is considered acceptable [22].

Delta values for metabolite concentrations (post intervention—pre intervention) for each individual were created and OPLS with effect projections (OPLS-EP) were used [23]. The advantage of OPLS-EP is that it takes into account individual variations in metabolite concentrations (equivalent to paired samples t-test) and can therefore provide information about the relative change in metabolite concentration from baseline to endpoint samples.

To select class discriminating variables of interest for annotation, variable importance in projection (VIP) scores >1.5 and loadings (w ≥± 0.1) were assessed. To adjust for multiple testing, a false discovery rate (FDR) with Benjamini-Hochberg approach [24] was applied; the variables included in our analyses represent approximately 150 metabolites, and with a critical value for FDR of 0.20, we considered p-values <0.016 to be statistically significant. All multivariate analyses were performed using SIMCA software v.15.0 (Umetrics AB, Umeå, Sweden).

## Results

**Fig 1:**
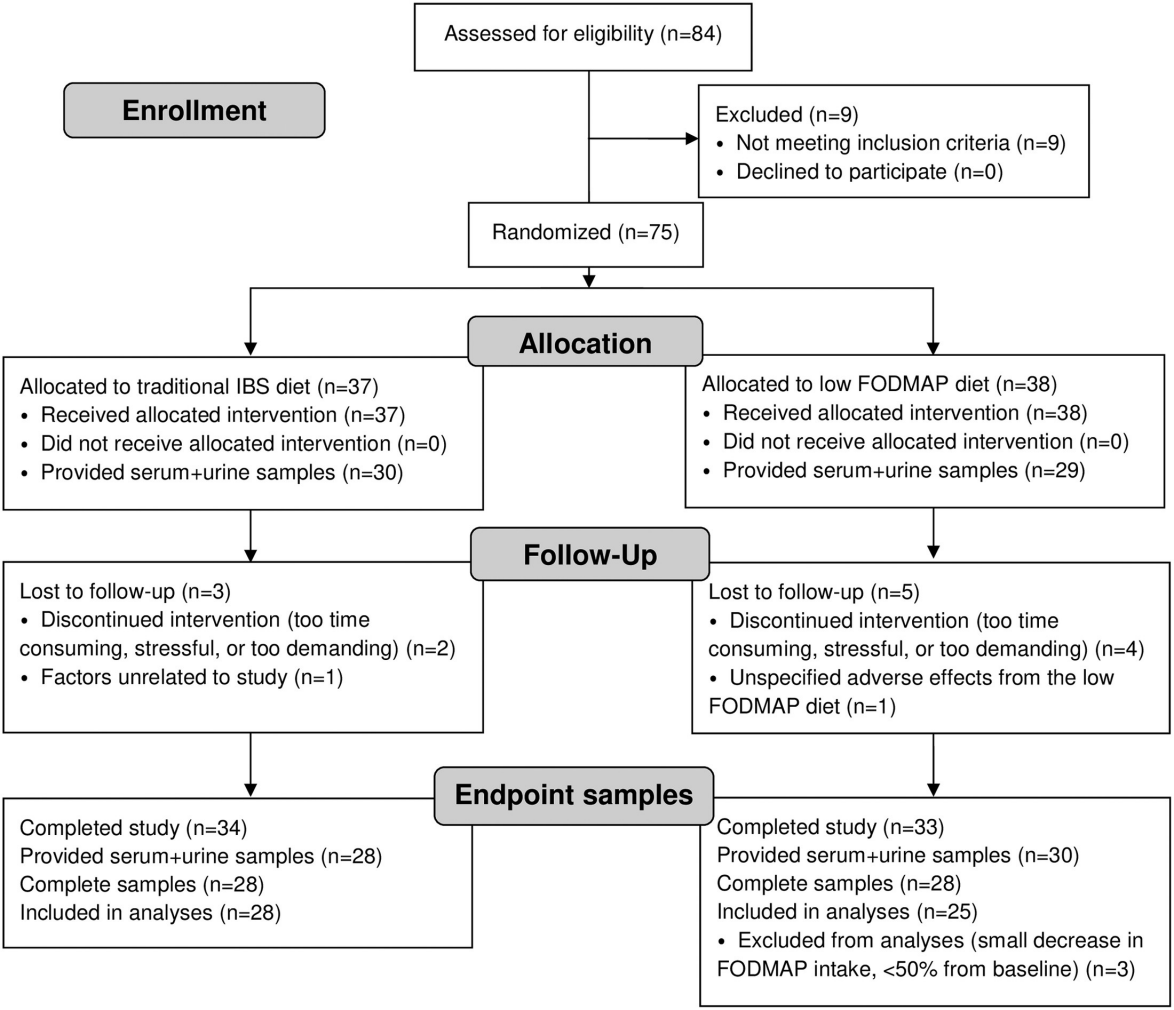
CONSORT flow diagram of study participants and sample collection.

In this post hoc analysis, we included all participants who provided serum and urine samples at both baseline and endpoint, giving a total of 56 participants. A flow chart of study participants and sample collection can be seen in Fig 1. Among the 28 participants allocated to the LFD we identified three individuals with very low baseline FODMAP intakes, who accordingly had made a relatively low reduction in FODMAP intake during the intervention (<50% of baseline values). To increase the chances of measuring a true effect of a FODMAP reduction, these three individuals were excluded from post-intervention analyses.

Clinical characteristics of the included study participants can be seen in Table 1.

**Table 1:** Clinical characteristics and demographics of study participants with complete data from pre- and post-intervention.

	Low FODMAP diet			Traditional IBS diet		
	all	responder^1^	non-responder	all	responder^1^	non-responder
	n = 28	n = 15	n = 13	n = 28	n = 14	n = 14
Age, *y (min*, *max)*	47.1 (21–69)	52.1 (33–69)	41.4 (21–66)	41.6 (18–68)	37.9 (18–68)	45.2 (20–68)
Sex, *n* (female/male)	23/5	14/1	9/4	23/5	13/1	10/4
BMI, *kg/m2 (min*, *max)*	24.8 (19.8–35.5)	23.7 (19.8–31.3)	25.9 (20.1–35.5)	23.7 (19.7–31.2)	23.3 (20.0–30.1)	24.1 (19.7–31.2)
IBS subtype, *n (%)*						
IBS-C	6 (21.4)	1 (6.7)	5 (38.5)	10 (35.7)	2 (14.3)	8 (57.1)
IBS-D	10 (35.7)	7 (46.7)	3 (23.1)	6 (21.4)	3 (21.4)	3 (21.4)
IBS-M	12 (42.9)	7 (46.7)	5 (38.5)	12 (42.9)	9 (64.3)	3 (21.4)
IBS symptom severity, *n (%)*						
moderate	9 (32.1)	4 (26.7)	5 (38.5)	14 (50)	6 (42.9)	8 (57.1)
severe	19 (67.9)	11 (73.3)	8 (61.5)	14 (50)	8 (57.1)	6 (42.9)

^1^Responder to diet intervention is defined as having ≥50 points reduction in IBS severity scoring system compared to baseline.

Abbreviations: IBS, irritable bowel syndrome; IBS-D, IBS with diarrhea; IBS-C, IBS with constipation; IBS-M, mixed type IBS; FODMAP, fermentable oligo-, di-, monosaccharides and polyols.

### Reported nutrient changes during the two interventions

Reported changes in energy, macronutrient, and FODMAP intake are summarized in S1 Table for both diets, and also divided into responders and non-responders to treatment. In the LFD, significant decreases in energy, all macronutrients (except alcohol) and FODMAP intake were seen. Responders to LFD reported significantly decreased intake in all nutrients, and non-responders to LFD reported decreased intake in energy, carbohydrate, fat, fiber, and FODMAP. In the TID, significant decreases in energy and fat intake were reported for the whole group. Responders to TID reported significantly decreased intake in energy and fat, while non-responders to TID did not report any significant changes in their energy nor nutrient intake.

### Metabolite patterns in baseline samples in relation to baseline characteristics

In the baseline samples (n = 59), we did not observe any clear clustering trends or patterns of metabolites related to age, gender, or reported dietary intake (macronutrients, fiber, and FODMAP) using PCA, in either serum (Table 2) or urine (Table 3) samples. OPLS in the baseline samples (n = 59), revealed age as the only significant predictor of metabolite patterns in both serum and urine (p = 0.0006 and p = 0.0004, respectively), and in serum also BMI (p = 0.007). OPLS models with reported baseline dietary intake, i.e. macronutrients, fiber, and FODMAP, yielded only weak models with no significant predictors of metabolite patterns.

**Table 2:** Multivariate models in serum from participants receiving a low FODMAP diet and traditional IBS diet.

	Model	Nr of Lv	*n*	R2X [cum]^1^	R2Y [cum]^2^	Q2 [cum]^3^	CV-ANOVA^4^ (p-value)	Permutation test (Q2)^5^
**1**	PCA-X	10	59	0.722	-	0.222	-	-
**2**	OPLS Y = age, BMI	2+2+0	59	0.414	0.688	0.37	-	-
**3**	OPLS Y = energy, protein, fat, carbohydrate, fiber, FODMAP	3+0+0	59	0.35	0.322	0.056	-	-
**4**	OPLS-EP LFD	1+2+0	25	0.481	0.887	0.364	0.151	0.364
**5**	OPLS-EP TID	1+1+0	28	0.37	0.601	0.239	0.146	0.239
**6**	OPLS-DA delta responders vs. non-responders LFD	1+0+0	25	0.212	0.324	0.0011	0.98	-0.0742
**7**	OPLS-DA delta responders vs. non-responders TID^6^	1+1+0	28	0.345	0.59	-0.835	1	-0.199

Abbreviations: FODMAP, fermentable oligo-, di-, monosaccharides and polyols; LFD, low FODMAP diet; LV, Latent Variables; TID, traditional IBS diet.

^1^Cumulative fraction of the sum of squares of X explained by the selected latent variables.

^2^Cumulative fraction of the sum of squares of Y explained by the selected latent variables.

^3^Cumulative fraction of the sum of squares of Y predicted by the selected latent variables.

^4^ANalysis Of VAriance testing of Cross-Validated predictive residuals.

^5^The intercept between real and random models, degree of overfit.

^6^This model was created by forcing the program to build a model with the two first components.

**Table 3:** Multivariate models in urine from participants receiving a low FODMAP diet and traditional IBS diet.

	Model	Nr of Lv	*n*	R2X [cum]^1^	R2Y [cum]^2^	Q2 [cum]^3^	CV-ANOVA^4^ (p-value)	Permutation test (Q2)^5^
**1**	PCA-X	3	59	0.478	-	0.202	-	-
**2**	OPLS Y = age, BMI, sex	2+1+0	59	0.431	0.373	0.182	-	-
**3**	OPLS Y = energy, protein, fat, carbohydrates, fiber, FODMAP	2+0+0	59	0.321	0.118	-0.0365	-	-
**4**	OPLS-EP LFD	1+2+0	25	0.392	0.899	0.423	0.07	0.423
**5**	OPLS-EP TID	1+0+0	28	0.153	0.313	-0.127	1	-0.127
**6**	OPLS-DA delta responders vs. non-responders LFD	1+4+0	25	0.58	0.941	0.447	0.29	-0.646
**7**	OPLS-DA delta responders vs. non-responders TID^6^	1+1+0	28	0.264	0.576	-1.04	1	-0.24

Abbreviations: FODMAP, fermentable oligo-, di-, monosaccharides and polyols; LFD, low FODMAP diet; LV, Latent Variables; TID, traditional IBS diet.

^1^Cumulative fraction of the sum of squares of X explained by the selected latent variables.

^2^Cumulative fraction of the sum of squares of Y explained by the selected latent variables.

^3^Cumulative fraction of the sum of squares of Y predicted by the selected latent variables.

^4^ANalysis Of VAriance testing of Cross-Validated predictive residuals.

^5^The intercept between real and random models, degree of overfit.

^6^This model was created by forcing the program to build a model with the two first components.

### Changes in metabolite patterns in relation to trial outcome

**Fig 2:**
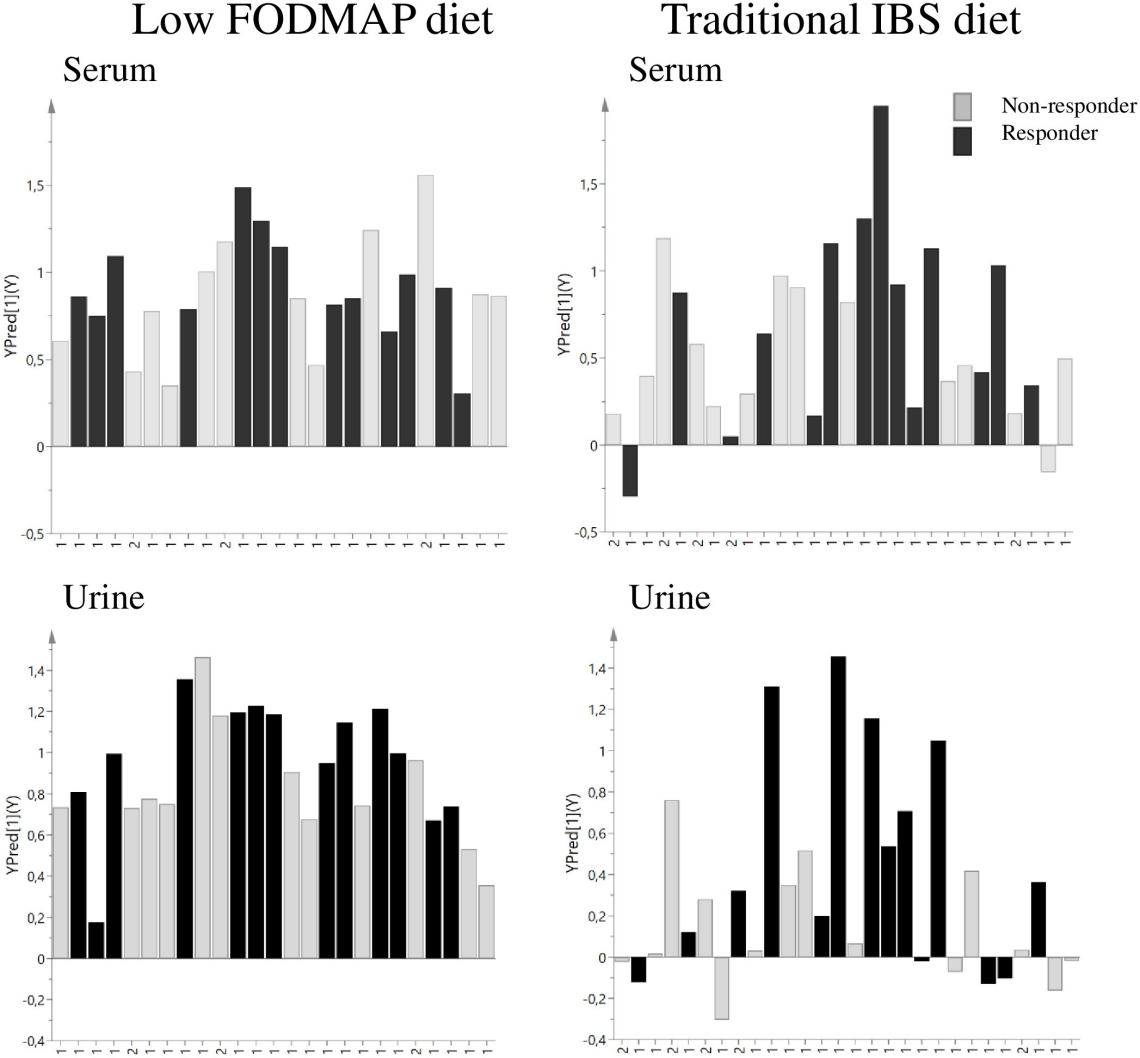
Observed response (YObs) in relation to predicted response (YPred) in the low FODMAP diet and traditional IBS diet in serum and urine samples, colored according to responders and non-responders and labeled 1 = women and 2 = men. YObs = 1 indicates a perfect match between observed and predicted values and variables with large positive or negative residuals are those differing with respect to the systematic structure captured by the model.

Changes in metabolite concentrations from baseline to endpoint within individuals were evaluated using OPLS-EP and visualized in Fig 2. A more uniform metabolite change was revealed in LFD compared to TID. In LFD, significant decreases in polyols, such as mannitol and sorbitol, were seen in the post-intervention samples compared to baseline, Table 4.

**Table 4:** Serum and urine metabolites responsible for class discrimination between baseline and endpoint samples, and between responders and non-responders in the low FODMAP diet.

Annotated metabolites	^1^H chemical shift region	Serum				Urine			
		Pre vs. post^1^		Δ Metabolites responders vs. non-responders^2^		Pre vs. post^1^		Δ Metabolites responders vs. non-responders^2^	
		*n* = 25		n = 25		n = 25		n = 25	
		post	p	resp.	p	post	p	resp.	p
Arginine, 2-Hydroxybutyrate	1.66328	-	-	↑	0.016*	-	-	-	-
Glutamine	2.15189	↓	<0.001*	-	-	-	-	-	-
Glucose	3.4596	↓	0.002*	↓	0.011*	-	-	-	-
Tyrosine, Ornithine	3.0559	-	-	↑	0.022	-	-	-	-
Mannitol, Sorbitol, Xylitol, Sucrose	3.93891	-	-	-	-	↓	<0.001*	-	-
Urea	5.76295	-	-	-	-	↑	0.045	-	-
Pantothenate	3.49325	-	-	-	-	-	-	↓	0.014*
Unknown	7.41651	-	-	-	-	-	-	↑	0.004*

*p-values <0.016 were considered statistically significant after correction for mass-testing.

Abbreviations: FODMAP, fermentable oligo-, di-, mono-saccharides and polyols; resp, responders; post, post-intervention; resp, responders.

^1^ Wilcoxon signed rank exact test for paired samples.

^2^ Mann-Whitney U test for unpaired samples.

When analyzing the changes in metabolite concentrations in relation to whether the participant had responded to dietary changes or not, also here a more distinct pattern was revealed in LFD compared to TID. Using OPLS-DA to separate responders vs. non-responders based on changes in metabolite concentrations, a five-component model with good fit and good predictive capacity (R^2^X = 0.58, Q^2^X = 0.447, Q^2^Y = 0.941) was yielded in urine for LFD. For TID, the dietary changes were probably too versatile to generate robust models. Thereafter, univariate analyses were applied on the metabolites that contributed most to the separation between responders and non-responders to diet intervention (i.e. delta values of metabolites). These analyses revealed that responders in LFD were characterized by significant decrease in glucose (serum) and pantothenate (urine) levels and significant increase in 2-hydroxybyturate (serum) and an unknown metabolite (urine), compared to non-responders (Table 4).

### Changes in single metabolites in relation to trial outcome

For the single metabolites that discriminated most between responders and non-responders in the OPLS-DA in LFD, logistic regression was applied to verify their significance. After adjustment for age and BMI, metabolites that showed borderline significant associations, given the FDR applied, were endpoint values of glucose (p = 0.049), pantothenate (p = 0.048) and change in glucose levels, i.e. delta values of glucose (p = 0.040), and pantothenate (p = 0.041).

## Discussion

This study, using biological samples from a randomized dietary trial in patients with IBS, demonstrated that alterations in serum and urinary metabolite concentrations can be detected using non-targeted NMR metabolomics. Also, changes in metabolite concentrations between responders and non-responders to an LFD can be differentiated. The major detectable differences in metabolite concentrations could be attributed to changes in intake of specific foods and/or reductions in energy and macronutrient intake.

A few previous studies have used metabolomics to elucidate the pathways in how an LFD alters symptoms in patients with IBS. One study, using mass spectroscopy to analyze urinary metabolites to compare a low and a high FODMAP diet, found that particularly three metabolites discriminated between the two diets; histamine, p-hydroxybenzoic acid and azelaic acid [14]. Histamine was reduced eightfold in the low compared to high FODMAP diet, suggesting that histamine plays a key role in pathophysiology of IBS. In this study, FODMAP intake was determined by a scoring system, making FODMAP intake difficult to quantify and to correlate to outcome measures. Our study did not reveal any major changes in histamine concentrations, but it might be that the large effect of altered protein and carbohydrate intake overshadowed these metabolites. Another study, using fecal volatile metabolites (VOC), did identify 15 features that predicted response to a LFD with high accuracy [25]. Altogether, metabolomics seems to be a promising and cost-effective tool to gather insight into the mechanistic actions of the LFD, but differences in biofluids used and analytical methods applied make comparisons of results difficult.

### Metabolite patterns in baseline samples

Using PCA, we did not observe any clear clustering patterns or trends in baseline metabolites in either urine or serum that could be related to reported dietary intake. This might be due to the heterogeneous study population with diversity in age, BMI, gender, and with different IBS subtypes, which all affect an individual’s phenotype and metabolic profile. Age also turned out to be a strong predictive component in our OPLS models, both in serum and urine, which seemed to overshadow the effect of the dietary components that were analyzed. A consequence of using fasting samples instead of postprandial samples is that we might capture more of the variation in personal characteristics (such as age and BMI) than in food intake, as some metabolites will have degraded during the overnight fasting. Some of the metabolites related to a favorable response to the dietary regime might therefore not be detected using fasting samples.

### Metabolite changes related to trial outcome

When using paired data (e.g. OPLS-EP) that account for individual variations, we were able to detect changes in the metabolite profiles that were related to the diets. In the TID, we noted a large variation in the individual metabolic response to treatment. A part of the explanation could be that the TID regime allows for more personalized modifications, resulting in varying metabolic responses to this diet. Precise and valid biomarkers of compliance to the TID likely do not exist.

The effect of the LFD was more uniform as seen in both serum and urine. Using univariate methods, we identified a significant decrease of polyols in urine in the LFD when comparing pre- and post-intervention samples. Polyols, such as mannitol and sorbitol, are sugar alcohols which are excluded from the diet as a part of the LFD regime. The decrease in urinary excretion of polyols in post-intervention samples is an indication of compliance to the dietary intervention and could potentially serve as a biomarker when studying polyol intake.

Multivariate models that discriminated responders from non-responders in the LFD were of acceptable quality and, using additional analyses, we were able to identify metabolites that differed significantly between responders and non-responders to treatment. We found that levels of glucose had decreased and 2-hydroxybyturate increased in serum during the LFD for responders compared to non-responders. 2-hydroxubyturate is an organic acid derived from alfa-ketobutyrate. Secretion of 2-hydroxybutyric acid is associated with lactic acidosis and ketoacidosis, sometimes attributed to an energy restrictive diet or a diet low in carbohydrates. Urinary metabolites that contributed most to the separation were pantothenate, which had decreased and was lower among responders to an LFD. Pantothenic acid is found in most foods consumed, but is especially prevalent in meat, legumes and whole grain cereals. The decrease in pantothenate could be a reflection of changes in foods consumed during the intervention, because legumes and whole grain cereals are both rich in fructo- and galacto-oligosaccharides; both excluded in the low FODMAP regime. An accompanying paper by Clevers et. al [26] showed that good compliance to the low FODMAP diet at food level led to a greater reduction in IBS-SSS, supporting our findings that metabolites were derived from FODMAP-rich foods.

### Strengths and limitations

This study was a post hoc analysis from our previously published intervention study comparing two diet regimes in patients with IBS [13], meaning that the results from this study must be interpreted as exploratory and hypothesis generating. A strength of this study is that the participants were well characterized using both biological samples and a wide range of validated and IBS-specific questionnaires. As patients were recruited both from our specialized GI unit at the hospital, but also through advertisement in the local newspaper, a broader range of patients with variation in IBS symptom severity could be ensured. Thus, these findings should be applicable to IBS patients in general. Optimally, when conducting multivariate analysis in metabolomics, one should validate the results with a validation set. However, as the sample size was small, we were unable to do this. External validation in a new and larger cohort is warranted to replicate these findings. Also, performing meal challenge test using predefined amounts of FODMAPs could further provide a deeper understanding of the metabolic pathways involved in treatment response.

### Conclusion

In this study we were able to distinguish metabolite patterns between individuals who responded to a LFD compared to those who did not. Metabolites that were identified as driving the separation between responders and non-responders may be attributed to certain foods or food groups. Some of these might also be derived from a restriction in energy and/or carbohydrate intake, providing new and interesting hypotheses for mechanistic actions related to response.

## Supporting information

S1 Checklist CONSORT 2010 checklist metabolomics. (DOC)

S1 Table Reported dietary changes (post—Pre) among responders and non-responders to treatment with low FODMAP diet and traditional IBS diet. (DOCX)

S1 File Forsknings plan KRIBS. (PDF)

S2 File Research plan. (DOCX)
