# Supplementary material for: Changes in serum and urinary metabolomic profile after a dietary intervention in patients with irritable bowel syndrome
Source: PLoS One. 2021 Oct 11;16(10):e0257331. doi: 10.1371/journal.pone.0257331 (PMC8504738; doi:10.1371/journal.pone.0257331)
Supplement: S1 Table — (DOCX) [file pone.0257331.s002.docx]

| Supplementary Table 1. Reported dietary changes (post – pre) among responders and non-responders to treatment with low FODMAP diet and traditional IBS diet. | | | | | | | | | | | | | | |
| --- | --- | --- | --- | --- | --- | --- | --- | --- | --- | --- | --- | --- | --- | --- |
|  | **Low FODMAP diet** | | | | | |  | **Traditional IBS diet** | | | | | |  |
|  | **all**  **n=28** | **pre vs. post** | **responders^2^ n= 15** | **pre vs. post** | **non-responders n= 13** | **pre vs. post** |  | **all**  **n=28** | **pre vs. post** | **responders^2^ n= 14** | **pre vs. post** | **non-responders n= 14** | **pre vs. post** | **P-value between diets^3^** |
|  | **mean** ± **SD** | **P-value**^1^ | **mean** ± **SD** | **P-value**^1^ | **mean** ± **SD** | **P-value**^1^ |  | **mean** ± **SD** | **P-value**^1^ | **mean** ± **SD** | **P-value**^1^ | **mean** ± **SD** | **P-value**^1^ |  |
| Energy (∆ kcal) | -380±290 | <0.001 | -380±290 | <0.001 | -379±300 | 0.001 |  | -206±468 | 0.031 | -364±527 | 0.023 | -36±338 | 0.70 | 0.09 |
| Protein (∆ gram) | -9.6±16.6 | 0.005 | -13.4±17.2 | 0.009 | -5.1±15.4 | 0.25 |  | -5.7±20.4 | 0.16 | -8.3±22.9 | 0.20 | -2.9±17.9 | 0.56 | 0.48 |
| Carbohydrate (∆ gram) | -39.3±47.0 | <0.001 | -35.3±41.5 | 0.005 | -44.0±53.9 | 0.012 |  | -6.6±58.7 | 0.56 | -21.2±73.1 | 0.30 | 9.1±34.1 | 0.36 | 0.016 |
| Fat (∆ gram) | -19.1±23.9 | <0.001 | -17.4±21.7 | 0.008 | -21.1±27.1 | 0.016 |  | -13.1±27.9 | 0.021 | -21.2±33.4 | 0.034 | -4.5±17.9 | 0.39 | 0.29 |
| Alcohol (∆ gram) | -1.4±6.5 | 0.251 | -4.5±4.2 | 0.001 | -2.1±6.9 | 0.284 |  | -3.8±10.3 | 0.07 | -4.5±11.6 | 0.174 | -3.0±9.2 | 0.26 | 0.61 |
| Fiber (∆ gram) | -4.4±5.8 | <0.001 | -4.1±5.0 | 0.007 | -4.8±6.9 | 0.028 |  | -0.2±8.8 | 0.90 | -0.7±7.8 | 0.76 | 0.3±10.0 | 0.92 | 0.001 |
| FODMAP (∆ gram) | -12.4±9.9 | <0.001 | -8.8±6.4 | <0.001 | -16.6±11.7 | <0.001 |  | -2.8±9.8 | 0.14 | -3.8±10.9 | 0.22 | -1.8±8.7 | 0.47 | <0.001 |
| Abbreviations: FODMAP, fermentable oligo,- di-, monosaccharides and polyols  ^1^ Paired student´s t-test comparing intake pre vs. post intervention  ^2^ Responder to diet intervention is defined as having ≥50 points reduction in IBS severity scoring system compared to baseline  ^3^ Student´s t-test comparing mean values post-intervention between diets | | | | | | | | | | | | | | |

Online Supporting Material
