# Supplementary material for: Changes in serum and urinary metabolomic profile after a dietary intervention in patients with irritable bowel syndrome
Source: PLoS One. 2021 Oct 11;16(10):e0257331. doi: 10.1371/journal.pone.0257331 (PMC8504738; doi:10.1371/journal.pone.0257331)
Supplement: S1 File — (PDF) [file pone.0257331.s003.pdf]

Forskningsplan

Jämförelse av låg-FODMAP-kost och traditionella kostråd vid IBS.

Lena Böhn, Stine Störsrud, Magnus Simrén, Hans Törnblom

Bakgrund

IBS (irritable bowel syndrome) är en funktionell mag-tarmsjukdom som kännetecknas av kronisk eller återkommande smärta och/eller obehag i buken i kombination med avföringsrubbning (1). Några av symtomen vid IBS är gasbesvär och buksvullnad (2). Framför allt kolhydrat- eller fettrik föda, samt kaffe, alkohol och kryddstark mat bidrar uppkomst till och förvärrande av dessa symtom. Vissa födoämnen som åstadkommer gasbildning är t.ex. mjölk, bröd, pasta, äpple, stenfrukter, baljväxter, kål och lök. Detta är livsmedel som innehåller stora mängder FODMAPs, dvs. fermenterbara oligo-, di-, monosackarider och sockeralkoholer (polyoler) (3). Att äta mat som innehåller låga mängder FODMAPs har visat sig vara effektivt för att minska gastrointestinala (GI) symtom (4).
De traditionella kostråden som ges vid IBS idag fokuserar mest på ätbeteende och på att minska de aktuella symtomen genom att ge specifika kostråd för dessa. Kostråden handlar då om att äta mindre måltider ofta och regelbundet samt att anpassa tillagningssätt och bearbetning av livsmedel för att på så sätt minska belastning på tarmen.
Det finns endast en studie som jämfört låg-FODMAP-kosten med de traditionella kostråden (4), och svenska undersökningar saknas helt.

Frågeställningar

- Att undersöka vilken kostregim som är mest effektiv av ”traditionell IBS-kost” eller ”låg-FODMAP-kost” för att lindra mag-tarmsymtom vid IBS.
- Att undersöka viken av ovanstående kostregim som har bäst följsamhet hos patienter med IBS.

Metod

Studien kommer att genomföras som en multicenter-studie (på Karolinska universitetssjukhuset i Stockholm, på Sabbatsbergs sjukhus (Aleris) i Stockholm och på Sahlgrenska universitetssjukhuset i Göteborg) och avser att inkludera totalt 70 patienter med IBS enligt ROME III (1).

Inklusionskriterier: Patienten ≥18 år skall ha diagnos IBS för att inkluderas i studien. De måste kunna läsa och skriva svenska. De måste också ha ≥175 poäng, dvs. ha måttlig-svår IBS, under 10-dagarsperioden som föregår studiestart enligt frågeformuläret IBS-SSS (se nedan). Om poängen inte uppnås exkluderas patienten vid besök 2.

Exklusionskriterier: Patienten får inte ha svår hjärt-, lever-, neurologisk eller psykiatrisk komorbiditet, inte heller någon allvarlig samtida mag-tarmsjukdom eller celiaki. Det får heller inte förekomma någon födoämnesallergi- eller intolerans.

Besök 1
Patienterna, som har remitterats till dietist för kostråd vid IBS på respektive sjukhus mag-tarmmottagning, tillfrågas om de vill ingå i studien.

De får muntlig samt skriftlig information om studien, dock ej några detaljer om respektive kost. Patienten skriver på informerat samtycke.

Basuppgifter om patienten (namn och kontaktuppgifter) samlas in.

Mellan besök 1 och 2
följer en screeningperiod då patienten fyller i formuläret BSF (se nedan) där avföringsfrekvens och –konsistens registreras under 10 dagar. En kostdagbok fylls också i onsdag-lördag (4 dagar) under screeningperioden. Patienten fyller i GSRS-IBS (se nedan) under periodens sista dag (dag -1). Patienten har vid besök 1 fått rör för avföring som skall samlas in under slutet av screeningperioden för analys av bakterieflora i tjocktarmen.

Besök 2- dag 0
Matdagboken samlas in och gås igenom med patienten för att kunna tolka den på bästa sätt gällande mängdangivelser och kostval.

Patienten fyller i frågeformuläret IBS-SSS (se nedan). Studieansvarig på respektive enhet kontrollerar genom svaret på IBS-SSS *att* patienten haft IBS-symtom i tillräckligt hög grad (≥175 poäng) under screeningperioden. Det får inte ha varit en symtomfri period, beroende på att det då kan vara svårt att se någon förändring av symtom för respektive kost. Om perioden varit symtomfri exkluderas således patienten.

Vid inklusion:

Patienten fyller därefter i ett häfte (”Besök 2”) med frågor om demografi samt frågeformulären VSI, PHQ-15, HAD och MFI-20 (se nedan).

Blodprov tas och urinprov lämnas för att undersöka näringsämnessammansättning i kroppen. Avföringsprovet tas emot.

Patienten randomiseras dubbelblint därefter att antingen ingå i låg-FODMAP-gruppen eller gruppen som får traditionella kostråd vid IBS (TRAD-gruppen) (35 patienter i respektive grupp);

- *Låg-FODMAP-kost (låg-FODMAP-gruppen).*

Patienten kommer få muntlig genomgång om kosten samt en lista (kost A) med ”otillåtna” livsmedel och ”tillåtna” livsmedel.

- *Traditionella kostråd vid IBS (TRAD-gruppen)*

Patienten kommer få muntlig genomgång samt skriftliga instruktioner (kost B).

Samtliga studiepatienter kommer att uppmanas att noggrant följa instruktionerna om vad och hur de skall äta under studiens 28 dagar och under denna tid skall patienterna också fylla i olika

frågeformulär vid specificerade tidpunkter. Frågeformulären finns samlade i fyra häften, ett häfte för varje vecka, som patienten får med sig tillsammans med tre svarskuvert. Respektive häfte skickas efter varje vecka till Mag-tarmlab, Sahlgrenska i Göteborg. Det sista (fjärde) häftet tas med till besök 3+avföringsprov.

Tjugofyratimmars kostanamnes görs slumpmässigt per telefon en gång i veckan. På detta sätt kan vi ta reda på om patienten följer kostråden, om det är svårt att hålla sig vid kosten och dessutom får patienten tillfälle att ställa frågor samt få tips och råd och livsmedel och tillagningsmetoder.

Besök 3

Patienten lämnar in häftet för vecka 4 (den sista veckans registrering) samt ifylld matdagbok och avföringsprovet lämnas in. Blodprov tas och urinprov lämnas.

Patienten får på plats fylla i ett häfte med frågeformulären HAD, MFI-20, VSI, PHQ-15 samt IBS-SSS. Patienten tillfrågas om hur studieperioden gått och uppmanas dessutom att fritt skriva ner egna tankar, idéer och synpunkter på ett blankt blad.

Frågeformulär

BSF, *Bristol Stool Form,* är en metod för att bedöma tarmfunktion. Patienten anger antal avföringstillfällen per dag samt kryssar för vilken avföringstyp 1-7 (hård till lös avföring) vid varje tillfälle (1).
Vid varje veckas sista BSF-fråga besvaras en extra fråga om buksmärta; *"Hur graderar du din totala buksmärta under de senaste 7 dagarna?".* Svaren graderas från betydligt förbättrad (2 poäng), måttligt förbättrad (1 poäng), oförändrad (0 poäng), måttligt försämrad (-1 poäng) till betydligt försämrad (-2 poäng). En förändring på minst 30 % från första frågetillfället (under screeningperioden) betraktas som positiv respektive negativ effekt på smärta (5).

IBS-SSS, *IBS Symptom Severity Score,* undersöker svårighetsgraden av IBS-symtom. Frågeformuläret bedömer intensiteten av IBS-symtom; frekvent och svårighetsgrad av buksmärta, svårighetsgrad av bloating, missnöje med avföringsvanor och påverkan på livet i allmänhet. Totalpoängen beräknas från dessa fem frågor och delas in i svårighetsgradsgrupper, <175 lätt IBS, ≥175-300 måttlig IBS, ≥300 svår IBS (6).

VSI, *The Visceral Sensitivity Index*, är ett formulär med 15 frågor som avgör graden av symtomspecifik ångest hos patienter med IBS. Totalpoängen löper från 0 poäng (ingen GI-specifik ångest) till maximalt 75 poäng (svår GI-specifik ångest)(7).

PHQ-15, *The Patient Health Questionnaire-15,* definierar graden av somatisering. Maxpoängen är 28 för män (exkluderat fråga om mens-problem) och 30 för kvinnor. Poäng 0-4 representerar minimal, 5-9 liten, 10-14 medium och ≥15 hög somatisk svårighetsgrad (8).

MFI-20, *The Multidimensional Fatigue Inventory -20*, som innehåller 20 påståenden angående fem olika aspekter av trötthet; allmän trötthet, fysisk trötthet, minskad aktivitet, minskad motivation och mental trötthet. Frågorna graderas från 1 till 5. Ett högre poäng innebär mer trötthet (9).

HAD, *Hospital Anxiety Depression*, för att bedöma graden av ångest och depression. Frågeformuläret består av 14 frågor med en 4-gradig Likertskala (0-3), med 7 frågor som specifikt gäller ångest och 7 frågor som specifikt rör depression. Ett värde ≥11 definierar patienten som "med" eller utan kliniskt signifikant ångest respektive depression (10).

GSRS-IBS, *Gastrointestinal Symptom Rating Scale,* för att avgöra graden av mag-tarmsymtom vid IBS. Formuläret består av 13 frågor angående svårighetsgrad (1, inga besvär alls *till* 7, väldigt svåra besvär) för mag-tarmsymtom som smärta, uppkördhet, diarré, förstoppning och mättnad. Då IBS-symtom varierar mycket över tid räknas medelpoängen ut för fyra veckor för varje bedömningsfråga (11).

Analyser

Jämförelser görs med avseende följsamhet till de båda kost-regimerna (kostanamneser samt kostdagbok).

Jämförelser görs också med avseende på symtom (GSRS-IBS, IBS-SSS, PHQ-15) och avföringsvanor (BSF), trötthet (MFI-20), samt ångest och/eller depression (HAD, VSI).

***Tabell 1.* Studieupplägg för båda grupperna oavsett kostintervention**

Tidpunkt			
Dag -11	***Besök 1*.**<br>Info + undertecknat samtycke.<br>Kontaktuppgifter till patient samlas in.		Rör för avföringsprov lämnas till patienten, tillsammans med matdagbok och symtomformulär.
Dag -10- -2	BSF (varje dag)		
Dag -1	BSF + smärtfråga<br>GSRS-IBS		
Onsdag-lördag	Matdagbok (4 dagar)		
Dag nära besök 2	Avföringsprov tas (vid ett tillfälle)		
Dag 0	***Besök 2***<br>Screeninghäftet samlas in och värderas.<br>Matdagbok samlas in och kontrolleras.<br>IBS-SSS besvaras och värderas.		
Vid inklusion:	Frågeformulär besvaras:<br>Demografi<br>VSI<br>PHQ-15<br>HAD<br>MFI-20	Blodprov tas.<br>Urinprov lämnas.<br>Avföringsprov tas emot.	Fyra häften med symtomformulär skickas med patienten tillsammans med tre svarskuvert, samt ett rör för avföringsprov som tas under sista veckan.
Dag 1-6	BSF (varje dag)	24-timmars-konstanamnes via telefon under veckan må-sön kl 8-20	
Dag 7	BSF + smärtfråga<br>GSRS-IBS		
Dag 8-13	BSF (varje dag)	24-timmars-konstanamnes via telefon under veckan må-sön kl 8-20	Dag 8: Patienten skickar in föregående veckas frågeformulär
Dag 14	BSF + smärtfråga<br>GSRS-IBS<br>IBS-SSS		
Dag 15-20	BSF (varje dag)	24-timmars-konstanamnes via telefon under veckan må-sön kl 8-20	Dag 15: Patienten skickar in föregående veckas frågeformulär
Dag 21	BSF + smärtfråga<br>GSRS-IBS		
Dag 22-27	BSF (varje dag)	24-timmars-konstanamnes via telefon under veckan må-sön kl 8-20	Dag 22: Patienten skickar in föregående veckas frågeformulär
Dag 28	BSF + smärtfråga<br>GSRS-IBS		
Onsdag-lördag	Matdagbok (4 dagar)		
Dag nära besök 2	Avföringsprov tas (vid ett tillfälle)		
Dag 29	***Besök 3***<br>VSI<br>PHQ-15<br>HAD<br>MFI-20<br>IBS-SSS	Blodprov tas.<br>Urinprov lämnas.<br>Avföringsprov tas emot.	Föregående veckas frågeformulär lämnas in av patienten.

Referenser

1. Longstreth GF, Thompson WG, Chey WD, Houghton LA, Mearin F, Spiller RC. Functional Bowel Disorders. Gastroenterology. 2006;130(5):1480-91.

2. Simrén M, Månsson A, Langkilde AM, Svedlund J, Abrahamsson H, Bengtsson U, et al. Food-Related Gastrointestinal Symptoms in the Irritable Bowel Syndrome. Digestion. 2001;63(2):108-15.

3. Gibson PR, Shepherd SJ. Evidence-based dietary management of functional gastrointestinal symptoms: The FODMAP approach. J Gastroenterol Hepatol. 2010;25(2):252-8.

4. Staudacher HM, Whelan K, Irving PM, Lomer MCE. Comparison of symptom response following advice for a diet low in fermentable carbohydrates (FODMAPs) versus standard dietary advice in patients with irritable bowel syndrome. J Hum Nutr Diet. 2011;24(5):487-95.

5. Guidance for Industry. Irritable Bowel Syndrome — Clinical Evaluation of Drugs for Treatment. 2012; Available from: www.fda.gov/downloads/Drugs/GuidanceComplianceRegulatoryInformation/Guidances/UCM205269.pdf

6. Francis CY, Morris J, Whorwell PJ. The irritable bowel severity scoring system: a simple method of monitoring irritable bowel syndrome and its progress. Aliment Pharmacol Ther. 1997;11(2):395-402.

7. Labus JS, Mayer EA, Chang L, Bolus R, Naliboff BD. The Central Role of Gastrointestinal-Specific Anxiety in Irritable Bowel Syndrome: Further Validation of the Visceral Sensitivity Index. Psychosomatic Medicine. 2007 January 1, 2007;69(1):89-98.

8. Kroenke K, Spitzer RL, Williams JBW. The PHQ-15: Validity of a New Measure for Evaluating the Severity of Somatic Symptoms. Psychosom Med. 2002 March 1, 2002;64(2):258-66.

9. Smets EMA, Garssen B, Bonke B, De Haes JCJM. The multidimensional Fatigue Inventory (MFI) psychometric qualities of an instrument to assess fatigue. Journal of Psychosomatic Research. 1995;39(3):315-25.

10. Zigmond AS, Snaith RP. The Hospital Anxiety and Depression Scale. Acta Psychiatr Scand. 1983;67(6):361-70.

11. Wiklund IK, Fullerton S, Hawkey CJ, Jones RH, Longstreth GF, Mayer EA, et al. An Irritable Bowel Syndrome-Specific Symptom Questionnaire: Development and Validation. Scand J Gastroenterol. 2003;38(9):947-54.
