# Supplementary material for: Changes in serum and urinary metabolomic profile after a dietary intervention in patients with irritable bowel syndrome
Source: PLoS One. 2021 Oct 11;16(10):e0257331. doi: 10.1371/journal.pone.0257331 (PMC8504738; doi:10.1371/journal.pone.0257331)
Supplement: S2 File — (DOCX) [file pone.0257331.s004.docx]

**Research plan**

**Comparison of a low FODMAP diet and traditional IBS dietary advice.**

**Background**

IBS (irritable bowel syndrome) is a functional gastrointestinal disorder characterized by chronic or recurrent pain and/or discomfort in the abdomen, in combination with abnormal bowel habits (1). Some of the symptoms of IBS include gas and bloating (2). In particular, carbohydrate- or high-fat foods, as well as coffee, alcohol and spicy foods, contribute to the onset and worsening of these symptoms. Some foods that cause flatulence are e.g. milk, bread, pasta, apple, stone fruits, legumes, cabbage and onions. These are foods that contain large amounts of FODMAPs, ie. fermentable oligo-, di-, monosaccharides and sugar alcohols (polyols) (3). Reducing intake of FODMAPs has been shown to be effective in alleviating gastrointestinal (GI) symptoms (4). The traditional dietary advice given to patients with IBS today focuses mainly on eating behavior, which include eating smaller meals on regular basis and adapting cooking methods, in order to reduce the load on the intestine. To date, there is only one study that has compared the low-FODMAP diet with the traditional dietary guidelines (4), and Swedish studies are completely lacking.

**Specific questions**

• To study which diet is most effective to relieve gastrointestinal symptoms in IBS; “traditional IBS diet” or a “low-FODMAP diet”.

• To examine the compliance to these abovementioned diet regimes.

**Method**

The study will be carried out as a multicenter study (at Karolinska University Hospital in Stockholm, at Sabbatsberg Hospital (Aleris) in Stockholm and at Sahlgrenska University Hospital in Gothenburg) and intends to include a total of 70 patients with IBS according to ROME III (1).

Inclusion criteria: Patient ≥18 years old must be diagnosed with IBS according to criteria. They must be able to read and write in Swedish. They must exhibit ≥175 points, ie. have moderate-severe symptoms of IBS according to the IBS-SSS questionnaire (see below), during the 10-day period preceding the randomization. If the score is not achieved, the patient is excluded at visit 2. Exclusion criteria: The patient must not have severe cardiac, hepatic, neurological or psychiatric comorbidity, nor any serious concomitant gastrointestinal disease or celiac disease. Also, no food allergies or intolerances are allowed.

**Visit 1**

The patients, who have been referred to a dietitian for dietary advice at each hospital's gastrointestinal clinic, are asked if they want to be included in the study. They receive oral and written information about the study, but no specific details about each diet. The patient signs written informed consent. Patients contact information is collected.

**Between visits 1 and 2** follows a screening period where the patient fills in the BSF (see below), in which stool frequency and consistency are registered for 10 days. A diet record is also filled in during Wednesday-Saturday (4 days) during the screening period. The patient completes GSRS-IBS (see below) during the last day of screening (day - 1). At visit 1, the patient received tubes for faecal samples to be sampled during the end of the screening period for analysis of gut microbiota composition.

**Visit 2-day 0**

The diet record is collected and reviewed together with the patient, in order to ascertain that estimate quantities and dietary choices are correct. The patient fills in the IBS-SSS questionnaire (see below). The study supervisor at each unit calculates the IBS-SSS, in order to evaluate if the patient exhibited IBS symptoms to a sufficient degree (≥175 points) during the screening period. It should not have been a symptom-free period, since it can lead to difficulties achieving any change in symptoms during intervention. If the screening period has been symptom-free, the patient is thus excluded.

**Patients who get randomized:**

The patient fills in a booklet ("Visit 2") with questions about demography and the questionnaires VSI, PHQ-15, HAD and MFI-20 (see below).

Blood samples are taken and urine samples are given to examine the nutritional status. Stool sample is received and frozen.

The study participant then gets double-blinded to be randomized in either the low-FODMAP group or the group receiving traditional dietary advice in IBS (the TRAD group) (35 patients in each group);

• Low-FODMAP diet (low-FODMAP group).

The patient will receive oral instructions of the diet and a list (diet A) of foods that should be omitted, and one list with permitted foods.

• Traditional dietary advice at IBS (TRAD group)

The patient will receive oral instructions and written instructions (diet B).

All study patients will be asked to carefully follow the instructions on what and how to eat during the 28 days of the study, and during this time the patients should also fill in different questionnaires at specified times. The questionnaires are collected in four booklets; one booklet for each week, which the patient receives together with three stamped envelopes. Each booklet is sent after each week to Mag-tarmlab, Sahlgrenska in Gothenburg. The last (fourth) booklet is brought to visit 3 + stool samples.

24-h diet recall is performed randomly by phone once a week. In this way, we can find out if the patient are compliant to the dietary advice, and also, the patient gets an opportunity to ask questions and get advice regarding food and cooking methods.

**Visit 3**

The patient submits the booklet for week 4 (the last week's registration) and the completed food diary and the stool sample. Blood and urine samples are taken.

The patient can fill in a booklet on site with the questionnaires HAD, MFI-20, VSI, PHQ-15 and IBS-SSS. Patient were finally asked about how they perceived the study period, and was also encouraged to freely write down their own thoughts, ideas and views on a blank sheet of paper.

**Questionnaires**

BSF, Bristol Stool Form, is a method for assessing bowel function. The patient notes the number of bowel movements per day and ticks for which bowel type 1-7 (hard to loose stools) they had at each occasion (1). At each week's last BSF question, an extra question about abdominal pain is answered; "How do you rate your total abdominal pain in the last 7 days?". The answers are graded from significantly improved (2 points), moderately improved (1 point), unchanged (0 points), moderately impaired (-1 points) to significantly impaired (-2 points). A change of at least 30% from the first question (during the screening period) is considered as a positive and negative effect on pain, respectively (5).

IBS-SSS, IBS Symptom Severity Score, examines the severity of IBS symptoms. The questionnaire assesses the intensity of IBS symptoms; frequent and severity of abdominal pain, severity of bloating, dissatisfaction with bowel habits and impact on life in general. The total score is calculated from these five questions and is divided into severity groups, <175 mild IBS, ≥175-300 moderate IBS, ≥300 severe IBS (6).

VSI, The Visceral Sensitivity Index, comprises of 15 questions that determine the degree of symptom-specific anxiety in patients with IBS. The total score ranges from 0 points (no GI-specific anxiety) to a maximum of 75 points (severe GI-specific anxiety) (7).

PHQ-15, The Patient Health Questionnaire-15, defines the degree of somatization. The maximum score is 28 for men (excluding menstrual problems) and 30 for women. Scores 0-4 represent minimal, 5-9 small, 10-14 medium and ≥15 high somatic severity (8).

MFI-20, The Multidimensional Fatigue Inventory -20, contains 20 statements regarding five different aspects of fatigue; general fatigue, physical fatigue, decreased activity, decreased motivation and mental fatigue. The questions are graded from 1 to 5. A higher score indicates more fatigue (9).

HAD, Hospital Anxiety Depression, assess the degree of anxiety and depression. The questionnaire consists of 14 questions with a 4-point Likert scale (0-3), with 7 questions that specifically concern anxiety and 7 questions that specifically relate to depression. A value ≥11 defines the patient as “with” or “without” clinically significant anxiety or depression (10).

GSRS-IBS, Gastrointestinal Symptom Rating Scale, determines the degree of gastrointestinal symptoms in IBS. The form consists of 13 questions regarding severity (1, no problems at all to 7, very severe problems) for gastrointestinal symptoms such as pain, bloating, diarrhea, constipation and satiety. As IBS symptoms vary greatly over time, the mean score is calculated for four weeks for each assessment question (11).

**Analyzes**

Comparisons are made with regard to compliance with the two diet regimens (diet recall and diet record).

Comparisons are also made with respect to symptoms (GSRS-IBS, IBS-SSS, PHQ-15) and bowel habits (BSF), fatigue (MFI-20), and anxiety and / or depression (HAD, VSI)

| *Timepoint* |  | | | |
| --- | --- | --- | --- | --- |
| Day -11 | ***Visit 1****.*  Information + signed consent  Contact information | |  | Tubes for fecal sampling, diet records and questionnaires are handled to the patient |
| Day -10- -2 | BSF (every day) | | | |
| Day -1 | BSF + pain  GSRS-IBS | | | |
| Wednesday-Saturday | Diet record (4 d) | | | |
| Day near visit 2 | Stool sample is taken | | | |
| Day 0 | ***Visit 2***  The screening booklet is collected and evaluated. Food record is collected and checked.  IBS-SSS is answered and evaluated. | | | |
| At inclusion: | Questionnaires  Demography | Blood, urine and stool samples collected. | | Four booklets for symptom registration are sent with the patient together with three envelopes, as well as a tube for stool sample to be taken during the last week. |
| Day 1-6 | BSF (every day) | 24-h diet recall by telephone  mon-sun between 8-20 h | | |
| Day 7 | BSF + pain  GSRS-IBS |  |  |  |
| Day 8-13 | BSF (every day) | 24-h diet recall | | Day 8: Patient mails this weeks questionnaire |
| Day 14 | BSF + pain GSRS-IBS  IBS-SSS |  |  |  |
| Day 15-20 | BSF (every day) | 24-h diet recall | | Day 15: Patient mails this weeks questionnaire |
| Day 21 | BSF + pain  GSRS-IBS |  |  |  |
| Day 22-27 | BSF (every day) | 24-h diet recall | | Day 22: Patient mails this weeks questionnaire |
| Day 28 | BSF + pain  GSRS-IBS |  |  |  |
| Wednesday-Saturday | Diet record (4 day) |  | |  |
| Day near visit 2 | Stool sample is taken |  | |  |
| Day 29 | ***Visit 3***  Questionnaires | Blood, urine and stool samples collected. | | Last week of questionnaires are handled in. |

***Tabel 1. Overview of study design***

Referenses

1. Longstreth GF, Thompson WG, Chey WD, Houghton LA, Mearin F, Spiller RC. Functional Bowel Disorders. Gastroenterology. 2006;130(5):1480-91.
2. Simrén M, Månsson A, Langkilde AM, Svedlund J, Abrahamsson H, Bengtsson U, et al. Food-Related Gastrointestinal Symptoms in the Irritable Bowel Syndrome. Digestion. 2001;63(2):108-15.
3. Gibson PR, Shepherd SJ. Evidence-based dietary management of functional gastrointestinal symptoms: The FODMAP approach. J Gastroenterol Hepatol. 2010;25(2):252-8.
4. Staudacher HM, Whelan K, Irving PM, Lomer MCE. Comparison of symptom response following advice for a diet low in fermentable carbohydrates (FODMAPs) versus standard dietary advice in patients with irritable bowel syndrome. J Hum Nutr Diet. 2011;24(5):487- 95.
5. Guidance for Industry. Irritable Bowel Syndrome — Clinical Evaluation of Drugs for Treatment. 2012; Available from: [www.fda.gov/downloads/Drugs/GuidanceComplianceRegulatoryInformation/Guidances/ UCM205269.pdf](http://www.fda.gov/downloads/Drugs/GuidanceComplianceRegulatoryInformation/Guidances/UCM205269.pdf)
6. Francis CY, Morris J, Whorwell PJ. The irritable bowel severity scoring system: a simple method of monitoring irritable bowel syndrome and its progress. Aliment Pharmacol Ther. 1997;11(2):395-402.
7. Labus JS, Mayer EA, Chang L, Bolus R, Naliboff BD. The Central Role of Gastrointestinal- Specific Anxiety in Irritable Bowel Syndrome: Further Validation of the Visceral Sensitivity Index. Psychosomatic Medicine. 2007 January 1, 2007;69(1):89-98.
8. Kroenke K, Spitzer RL, Williams JBW. The PHQ-15: Validity of a New Measure for Evaluating the Severity of Somatic Symptoms. Psychosom Med. 2002 March 1, 2002;64(2):258-66.
9. Smets EMA, Garssen B, Bonke B, De Haes JCJM. The multidimensional Fatigue Inventory (MFI) psychometric qualities of an instrument to assess fatigue. Journal of Psychosomatic Research. 1995;39(3):315-25.
10. Zigmond AS, Snaith RP. The Hospital Anxiety and Depression Scale. Acta Psychiatr Scand. 1983;67(6):361-70.
11. Wiklund IK, Fullerton S, Hawkey CJ, Jones RH, Longstreth GF, Mayer EA, et al. An Irritable Bowel Syndrome-Specific Symptom Questionnaire: Development and Validation. Scand J Gastroenterol. 2003;38(9):947-54.
